# Effects of a demand-led evidence briefing service on the uptake and use of research evidence by commissioners of health services: protocol for a controlled before and after study

**DOI:** 10.1186/s13012-014-0199-4

**Published:** 2015-01-09

**Authors:** Paul M Wilson, Kate Farley, Carl Thompson, Duncan Chambers, Liz Bickerdike, Ian S Watt, Mark Lambert, Rhiannon Turner

**Affiliations:** Manchester Business School, University of Manchester, Manchester, M15 6 PB UK; Department of Health Sciences, University of York, York, YO10 5DD UK; School of Health and Related Research, University of Sheffield, Sheffield, S10 2TN UK; Centre for Reviews and Dissemination, University of York, York, YO10 5DD UK; Public Heath England North East Centre, Newcastle upon Tyne, NE15 8NY UK; School of Psychology, Queen’s University Belfast, Belfast, BT7 1NN UK

## Abstract

**Background:**

Clinical Commissioning Groups (CCGs) are mandated to use research evidence effectively to ensure optimum use of resources by the National Health Service (NHS), both in accelerating innovation and in stopping the use of less effective practices and models of service delivery. We intend to evaluate whether access to a demand-led evidence service improves uptake and use of research evidence by NHS commissioners compared with less intensive and less targeted alternatives.

**Methods/design:**

This is a controlled before and after study involving CCGs in the North of England. Participating CCGs will receive one of three interventions to support the use of research evidence in their decision-making: 1) consulting plus responsive push of tailored evidence; 2) consulting plus an unsolicited push of non-tailored evidence; or 3) standard service unsolicited push of non-tailored evidence. Our primary outcome will be changed at 12 months from baseline of a CCGs ability to acquire, assess, adapt and apply research evidence to support decision-making. Secondary outcomes will measure individual clinical leads and managers’ intentions to use research evidence in decision making. Documentary evidence of the use of the outputs of the service will be sought. A process evaluation will evaluate the nature and success of the interactions both within the sites and between commissioners and researchers delivering the service.

**Discussion:**

The proposed research will generate new knowledge of direct relevance and value to the NHS. The findings will help to clarify which elements of the service are of value in promoting the use of research evidence. Those involved in NHS commissioning will be able to use the results to inform how best to build the infrastructure they need to acquire, assess, adapt and apply research evidence to support decision-making and to fulfil their statutory duties under the Health and Social Care Act.

**Electronic supplementary material:**

The online version of this article (doi:10.1186/s13012-014-0199-4) contains supplementary material, which is available to authorized users.

## Background

The National Health Service (NHS) in England is facing severe funding constraints both now and in the medium term. The forecast reduction in resources will place considerable pressures on its organisations and staff. In challenging times, it has been proposed that the greatest potential savings may be found by increasing efficiency and reducing variations in clinical practices [1]. To do this well, those commissioning health services need to be fully aware of the strength of the underlying evidence for interventions or ways of working that promise to deliver more value from the finite resources available [2].

The 2012 Health and Social Care Act has brought about a major shift in the commissioning landscape in England [3]. The Act has mandated the national and local entities, NHS England and Clinical Commissioning Groups (CCGs) to promote ‘the use of evidence obtained from research’.

Traditionally, public health specialists have supported and facilitated the use of research evidence in a commissioning context [4,5]. But with its relocation to local authorities, this constituency now has a more limited role in commissioning. The responsibility for developing the absorptive capacity [6,7] of CCGs to recognise and understand valuable research based knowledge is less clear.

Significant investment has been made in the production of research on the effectiveness and cost effectiveness of interventions to inform decisions and choices. An initiative aiming to enhance uptake of this knowledge to increase efficiency, reduce practice variations and to ensure best use of finite resources was developed as part of the National Institute for Healthcare Research (NIHR) Collaboration for Leadership in Applied Health Research and Care for Leeds, York and Bradford [8] The service works with local NHS commissioners and senior managers in provider Trusts to provide research-based answers to questions they raise. The service summarises and translates existing sources of synthesised, quality-assessed evidence (primarily systematic reviews and economic evaluations) to the local context. Topics addressed have included evidence to inform service reorganisation for adolescents with eating disorders [9], to support nurse/doctor role substitution and the introduction of integrated care pathways in mental health settings [10].

The service approach is both consultative [11] and responsive and operationalises a methodological framework [12] that involves clarifying the problem and framing the question to be addressed. The evidence briefings generated as part of this process summarise the quality and the strength of existing systematic reviews and economic evaluations but go beyond effectiveness and cost effectiveness to consider local applicability, implications relating to service delivery, resource use, implementation and equity.

A key feature of the service has been interaction and regular contact (face to face and email) between researchers and a range of clinicians, commissioners and NHS mangers to discuss and formulate questions that require a more considered response and to then produce briefings and discuss their implications. Although feedback has been uniformly positive to date, this service is developmental and has yet to be formally evaluated.

Interactions between researchers and NHS managers might be expected to facilitate the ongoing use of research knowledge in decision-making [13–17]. How best to do this [18] and the time and resource costs required for both sides are less clear. What is clear is that the benefit of interactions between managers and researchers is theoretically grounded. Specifically, ongoing, positive intergroup contact [19] can be effective at generating positive relations between the members of two parties where there is institutional support, where there is equal status between those involved, and where there is cooperation in order to achieve a common goal [20]. Contact has most benefit if those involved identify both with their own group (e.g. researchers or managers) and the overarching organisation to which they both belong [21].

Given the resource-intensive nature of the evidence briefing service, we need to establish how much value is added by additional support from researchers over alternative or more basic dissemination approaches. A recent systematic review described resources aimed at making the results of systematic reviews more accessible to health-care decision-makers [22]. A variety of resources were identified but few were evaluated, giving little insight into their impact on decision-making. As such, this study aims to add insight by evaluating the impact of a real-time, consultative knowledge translation service provided by researchers in response to real-life uncertainties identified by NHS commissioners.

## Methods

Primary research question

- Does access to a demand-led knowledge translation service improve uptake and use of research evidence by NHS commissioners compared with less intensive and less targeted alternatives?

Secondary research questions

- Do evidence briefings (summaries of synthesised research evidence with additional contextual information and implications for decision-making) tailored to specific local contexts inform decision-making in other CCGs?
- Does contact between researchers and NHS commissioners increase use of research evidence?

### Setting and participants

CCGs from one geographical area in the North of England will be contacted and told the nature of the study and then invited to participate. We have deliberately opted to conduct the study in a geographical area not contaminated by either our own earlier developmental work or other Collaboration for Leadership in Applied Health Research and Care (CLAHRC) related activity. Those CCGs that agree to participate will be asked to provide details of all governing body and executive members, clinical leads and any other individuals deemed as being involved in commissioning decision-making processes. These individuals will then be contacted by the evaluation team, told the purpose of the study and then invited to complete baseline and follow-up assessments. We anticipate each intervention arm will include at least two CCGs.

### Baseline and follow-up assessment

We will collect data for two outcome measures at baseline and post-intervention, after the 12-month intervention period has been completed.

The survey instrument (Additional file 1) will collect four sets of information. Section A is based on a tool originally devised by the Canadian Health Services Research Foundation [23,24] and then modified by the SUPPORT Collaboration [25]. This section assesses the organisations’ ability to acquire, assess, adapt and apply research evidence to support decision-making. Section B is a modified version of a tool [26,27] based on the theory of planned behaviour [28]. This will measure the intentions of individual CCG staff to use research evidence in their decision-making.

Section C is designed to evaluate the changes to the nature of the (proposed) interactions, both within the participating sites and between commissioners and researchers. Participants will be asked how much contact they have had with researchers in their job (quantity), and the success of the interaction (quality), using an existing modified measure [29]. This section will also include questions regarding the extent to which the interactions were perceived as friendly and cooperative, as helping to achieve the goals of both managers *and* researchers. The extent to which those involved in the interaction is perceived as being on an equal footing, without either group dominating, and the extent to which the contact is perceived as being supported by the CCGs, and the NHS more generally, will also be examined. Participants will also be asked to indicate the extent to which their status as an NHS manager/lead is important to them (in-group identification) and to what extent they see themselves and researchers as part of one overarching group committed to achieving the same things (superordinate identification). In addition, we will include measures of perceptions of researchers in general using a generalised intergroup attitude scale [30]. Section D captures information on individual respondent characteristics (for example, previous experience of doing research and self-reported uptake of new ideas) will be collected to help us to understand variation in responses.

Individual responses from each CCG will complete the survey and scores of all responses will be aggregated to represent each participating CCG. We will also be interested in variation in scores within each CCG.

A second survey based on Section A will be used to collect data from all English CCGs. This will include only the first outcome measure, and this will be delivered at baseline and then again post-intervention. As CCGs are new and evolving entities, we need to be able to determine if any changes viewed from baseline are linked to the intervention(s) and are not just a consequence of the development of the CCG(s) over the course of the study. To guard against this maturation bias, and to test the generalizability of findings, we will administer the Canadian Health Services Research Foundation (CHSRF) instrument to all English CCGs to assess their organisational ability to acquire, assess, adapt and apply research evidence to support decision-making. The most senior manager (chief operating officer or chief clinical officer) of each CCG will be contacted and asked to complete the CHSRF instrument on behalf of their organisation.

Both survey instruments will be sent by email to identified participants via an embedded URL. The online questionnaire will be hosted by SurveyMonkey website (http://www.surveymonkey.com). Reminder emails will be sent out to non-respondents at 2 3, and 4 weeks. A paper version of the questionnaire will also be posted out and phone call reminders will be utilised if required.

### Interventions

Participating CCGs will receive one of the three interventions aimed at supporting the use of research evidence in their decision-making: 1) consulting plus responsive push of tailored evidence; 2) consulting plus an unsolicited push of non-tailored evidence; or 3) ‘standard’ service unsolicited push of non-tailored evidence. The intervention phase will run from April 2014 to May 2015. The extent to which the CCGs seek and receive the interventions on offer will be determined by the CCGs themselves.

1. Consulting plus responsive push of tailored evidence
  After initial relationship building, participating CCGs in this arm will receive access to an evidence briefing service provided by the Centre for Reviews and Dissemination (CRD). The CRD team will synthesise existing evidence together with relevant contextual data to produce six to eight tailored evidence briefings on specified topics. Based on developmental work undertaken as part of the NIHR CLAHRC for Leeds, York and Bradford, we have resourced the project so that we can respond to six to eight key substantive issues during the intervention phase.
  The CRD intervention team will provide regular advice and support on how to seek solutions from existing evidence resources, question framing and prioritisation. Advice and support will be both reactive and proactive and will be delivered via telephone, email and face to face. Contact initiated by the CRD team will be made on at least a monthly basis and is expected to include: discussion of progress on ongoing topics, identification of further evidence needs and discussion of any issues around use of evidence. The team will also be alerting to new systematic reviews and other synthesised evidence relevant to CCG priorities.
  The intervention team will also offer to provide training on how to acquire, assess, adapt and apply synthesised existing evidence. Sessions will be based on the approach developed by the CRD service [12] and will be drawn upon the *Tools for Policymakers* developed by the Support Collaboration (see www.health-policy-systems.com/). Training to be provided will depend on the needs on the CCG participants but is likely to cover: question framing, priority setting, identifying and appraising systematic review evidence, assessing uncertainty and generalizability.
2. Consulting plus an unsolicited push of non-tailored evidence
  Participating CCGs in this arm will receive the access to regular advice and support from CRD as those in intervention 1). However, CRD will not produce evidence briefings tailored to the local CCGs context and their specified decisions but will instead disseminate the evidence briefings generated in intervention 1) with any area-specific contextual information removed; thus an intervention comprising consulting plus an unsolicited push of non-tailored evidence.
3. ‘Standard service’ unsolicited push of non-tailored evidence
  The third intervention constitutes a ‘standard service’ control arm. In this, CRD will disseminate the evidence briefings generated in intervention 1) and any other non-tailored briefings produced by CRD over the intervention period; thus, an unsolicited push of non-tailored evidence.

### Analysis

Baseline and follow-up assessment will be undertaken by a separate evaluation team. The CRD intervention team members delivering the intervention components will be blinded from data and analysis.

The evaluation team will use ANOVAs to examine whether participants in the intervention conditions perceive themselves as experiencing more positive contact experiences and more positive attitude towards researchers over time in the intervention conditions compared to the control condition.

The primary analysis will measure the impact of study interventions on two main outcomes at two times points. The key dependent variable will be the perceived organisational capacity to use research evidence, but we will also measure the impact of interventions upon our second outcome of reported research use. These will be treated as continuous variables and for each we will calculate the overall mean score, any sub scale means and related standard deviations at two time points (pre- and post-intervention) and within four case sites. Secondary analysis will assess interactions between the intervention received and three further continuous independent variables measuring individual demographic characteristics and the quality and frequency of contact, upon the two outcome measures.

For each of these variables, we will conduct a two-way repeated measures ANOVA with two within-subject factors (case site [as a proxy for the model of evidence briefing service received] and time period [pre- and post-intervention]). SPSS version 21 ‘GLM’ analysis procedure will be used.

If data subsequently warrants more complex multivariate analysis, we will explore the possibilities with a departmental statistician and the scientific advisory group. Where measures are non-normal, we will transform the data (logarithmically) where necessary and possible. Analysis will be undertaken using SPSS (version 20) and STATA statistical packages.

At follow-up, we will provide an opportunity in the questionnaire for CCGs senior officers to provide additional information that they think has changed, in their organisation in the past 12 months. We will code the responses to these questions thematically as well as response/no response and enter the response as a covariate in any analysis of variance conducted to examine for systematic patterns of response amongst the variable categories.

Where attrition between baseline and one-year follow-up is an issue, multiple imputation (MI) techniques will be employed. In addition, we will use guidance on interpreting effect sizes in before and after studies to examine the significance of any changes [31].

### Documentary evidence of the use of research in decision-making

We aim to identify and understand the ways in which research evidence is employed by each organisation through analysis of decision-making records. Selection of relevant documents will be conducted through review of a snapshot (one calendar month) of all documents produced by the CCG commissioning decision-making bodies. The evaluation team will then identify relevant documents to include over the course of the study. The precise nature of the analysis will be led by the content of the documents available; however, it is anticipated that the following qualitative analysis will be conducted. This will explore the integration of the evidence briefings service within decision-making processes, how research evidence in general is used by each organisation and how these change over time. Using a framework approach [32] and using NVivo software (NVivo qualitative data analysis software; QSR International Pty Ltd. Version 10, 2012), documents will be thematically coded to capture the ways in which research evidence has been used in the decision-making process. In order to identify changes in the use of evidence over time, documents will be categorised in quarterly periods with themes being compared across time periods.

### Qualitative interviews

In-depth qualitative interviews with governing and executive body members in participating case sites will be conducted. These will explore perceptions of the use of research evidence locally, their experiences of the process of the evidence briefing service and study processes as well as any unanticipated consequences of the work. This will add richness and depth to our quantitative measures and help us to understand the study results. The purposive sampling criteria will seek to include CCG who have had contact with the intervention team.

The framework approach [32] to analysis will again be applied to interview data. Deductive and inductive themes will be generated and interpreted using Atlas-TI (www.atlasti.com) to organise and manage the data. Member validation will be employed with all participating CCGs in each of the local health economies. In particular, it is anticipated that themes relating to the following areas will be explored: knowledge of and perceptions of the aims and expectations of the evidence briefing service received by the case site; expectations of and perceived impact of the evidence briefing service delivered in the CCG; attitudes to researcher evidence and how these relate to the evidence briefing service received; and perceptions of the use of research evidence locally.

### Data integration

This is a mixed methods study using a sequential explanatory strategy. The primary point of data integration will be the analysis stage in which themes generated by qualitative analysis will be used to help us to understand variation in quantitative outcomes. During this process, data will be integrated in three ways. Firstly, interviews will be categorised according to the intervention received and differences in the themes generated by each interview will be compared and contrasted across case sites. Second, individual interviews will also be categorised according to the participant’s survey responses to questions about relationships with researchers. Finally, themes generated by interviews will be compared with those arising from documentary evidence to identify any conflict or consistency between local perceptions of the use of evidence and recorded use of evidence.

### Ethical and consent issues

No ethical issues are anticipated as a result of this study. None of the interventions involves any direct risks or burdens to the CCGs involved. This study has been granted ethical permission by the Department of Health Sciences, University of York Research Ethics Board. Appropriate research governance approval has been obtained.

Organisational level consent granting permission to contact staff will be obtained from each participating CCG. Participants will have the opportunity to discuss any aspect of the study and their involvement in it with the research team at any stage of the study. Completion of questionnaires by individuals will be anonymous and other members of each CCG will not be informed of individual participation.

Informed consent will be sought from those participants approached for interview as part of the process evaluation. Those that indicate that they are not willing to be interviewed will be deemed not to have given their consent. For those participants that indicate that they are willing to be interviewed, we will send written confirmation of the arrangements and at this point, ask interviewees to provide written confirmation of their consent to participate.

## Discussion

The study has the potential to benefit NHS organisations by helping them make better use of existing synthesised research evidence to support their decision-making. This research is timely because of the current and future need to use research evidence effectively to ensure optimum use of resources by the NHS, both in accelerating innovation and in stopping the use of less effective practices and models of service delivery.

The research will also address the gap in translation of evidence into NHS practice identified in the Cooksey report [33] and the need for accelerated innovation highlighted by the Carruthers report [34]. The intervention phase of the research will generate new knowledge of direct relevance and value to CCGs. The service could result in more rapid decisions to adopt new treatments or models of service delivery; greater transparency of decision-making; and efficiency gains generated by evidence-informed decisions to disinvest existing services in favour of more cost-effective alternatives.

We have adopted a pragmatic comparative mixed methods research design (combining positivism and interpretivism). The approach will enable triangulation of data and add a depth of understanding to the impact of the service being offered. Whilst details of the underlying context and specific decisions may vary, findings about the impact of the demand-led service are likely to be broadly generalizable to other similar decision-making bodies.

The study should help to clarify which elements of the service are of value in promoting the use of research evidence and are worth pursuing further. For example, if the interaction and synthesis elements of the delivery model prove to have value, there is potential for developing a standardised service on a more expanded basis. This in turn will present opportunities for evaluations that utilise more robust designs or that seek to explore the service delivery setting and or its applicability in other geographical jurisdictions.

## Conclusion

The proposed research addresses a problem that faces a wide variety of health care organisations, namely how to best build the infrastructure they need to acquire, assess, adapt and apply research evidence to support their decision-making. For CCGs and the NHS England, this includes fulfilling their statutory duties under the Health and Social Care Act 2012.

## Additional file

Additional file 1:
**Clinical Commissioning Groups’ use of research evidence.**

## Abbreviations

CCG: Clinical Commissioning Group
CLAHRC: Collaboration for Leadership in Applied Health Research and Care
NHS: National Health Service
NIHR: National Institute for Healthcare Research
