# Supplementary material for: Effects of a demand-led evidence briefing service on the uptake and use of research evidence by commissioners of health services: protocol for a controlled before and after study
Source: Implement Sci. 2015 Jan 9;10:7. doi: 10.1186/s13012-014-0199-4 (PMC4296534; doi:10.1186/s13012-014-0199-4)
Supplement: Additional file 1: — Clinical Commissioning Groups’ use of research evidence. [file 13012_2014_199_MOESM1_ESM.pdf]

University of York
Centre for Reviews and Dissemination

NHS
National Institute for Health Research

Clinical Commissioning Groups' use of Research Evidence

We are exploring the use of research evidence by Clinical Commissioning Groups. You are being asked to participate because you are a member of a Clinical Commissioning Group governing body that has agreed to be part of this research study. We want your perspective on your organisation's decision-making processes.

We appreciate that you have many demands on your time but it is important that as many CCG members as possible complete and return the questionnaire. There are four sections in this questionnaire. **Each section does not need to be completed in one sitting so please feel free to complete each section individually at your convenience**. However, it is important that all four sections are completed in full in order for your data to be useful. All responses are on short scales, none require any written responses.

If you are ready to go, read and (if you agree), tick the two consent boxes below. Return the questionnaire to the research team at the University of York using the pre-paid envelope provided.

I have read and understood the information sheet provided ☐

I understand that by completing and returning this questionnaire I am giving my permission for the data I provide to be analysed and reported by the research team at the University of York ☐

Section 1 Organisational Capacity for Using Research Evidence

This first section is interested in the way in which *you* think *your* Clinical Commissioning Group uses research evidence. Please respond to each question by circling the **single number** that most closely fits your view. Please note the scales differ between each set of questions.

Questions 1-5

	Strongly disagree	Disagree	Neither agree nor disagree	Agree	Strongly agree
We have **skilled research staff**.	1	2	3	4	5
We have **arrangements with external experts** who search for research, monitor research, or do research for us.	1	2	3	4	5
Our staff have...					
...**enough time** for research.	1	2	3	4	5
... the incentive to do research (it is used in our decision-making).	1	2	3	4	5
... **the resources** to do research.	1	2	3	4	5

6-8 We look for research in...

	Don't do	Do poorly	Do inconsistently	Do with some consistency	Do well
...**journals** (that is by subscription, internet, or library access).	1	2	3	4	5
...**non-journal reports** by library, internet access, or direct mailing from organisations such as the Department of Health or King's Fund.	1	2	3	4	5
... **databases** by subscription or Internet access, such as the Cochrane Collaboration, DARE, and citation indices (PUBMED).	1	2	3	4	5

9-12

	Don't do	Do poorly	Do inconsistently	Do with some consistency	Do well
We **look for information** on **web sites** (that collate and/or evaluate sources) such as Clinical Evidence.	1	2	3	4	5
We **work with researchers** through formal and informal networking meetings with our staff.	1	2	3	4	5

We **get involved with researchers** as a host, decision-maker partner, or sponsor.	1	2	3	4	5
We **learn from peers** through informal and formal networks to exchange ideas, experiences, and best practices.	1	2	3	4	5

13-15 Our staff...

	Strongly disagree	Disagree	Neither agree nor disagree	Agree	Strongly agree
... have **critical appraisal skills and tools** for evaluating the **quality** of methodology used in research.	1	2	3	4	5
... have the **critical appraisal skills** to evaluate the **reliability** of specific research by identifying related evidence and comparing methods and results.	1	2	3	4	5
... can relate **research to our organisation** and point out similarities and differences.	1	2	3	4	5

16-17 Our CCG has arrangements with external experts...

	Strongly disagree	Disagree	Neither agree nor disagree	Agree	Strongly agree
... who use **critical appraisal skills and tools** to assess methodology and evidence reliability, and to compare methods and results.	1	2	3	4	5
... to **identify the relevant similarities and differences between** what we do and what the research says.	1	2	3	4	5

18-21 Our CCG has enough skilled staff with time, incentives, and resources who use research communication skills to...

	Strongly disagree	Disagree	Neither agree nor disagree	Agree	Strongly agree
... **present research** results concisely and in accessible language.	1	2	3	4	5
... **synthesize all relevant research**, along with information and analysis from other sources.	1	2	3	4	5
... **link research** results to key issues facing our decision makers.	1	2	3	4	5
... **provide recommended actions** to our decision makers.	1	2	3	4	5

22-25 Our CCG has arrangements with **external experts** who use research communication skills to...

	Strongly disagree	Disagree	Neither agree nor disagree	Agree	Strongly agree
... **present research** results concisely and in accessible language.	1	2	3	4	5
... **synthesize all relevant research**, along with information and analyses from other sources.	1	2	3	4	5
... **link research** results to key issues facing our decision makers.	1	2	3	4	5
...**provide recommended actions** to our decision makers.	1	2	3	4	5

26-32

	Strongly disagree	Disagree	Neither agree nor disagree	Agree	Strongly agree
Using **research is a priority in our CCG.**	1	2	3	4	5
Our CCG has committed **resources** to ensure **research is accessed, adapted, and applied in making decisions.**	1	2	3	4	5
Our CCG ensures **staff** are **involved in discussions on how research evidence relates** to our main goals.	1	2	3	4	5
The **management** of our CCG has clearly **communicated our strategy and priorities** so that those creating or monitoring research know what is needed in support of our goals.	1	2	3	4	5
We **communicate internally** in a way that ensures there is information exchanged across the entire organisation.	1	2	3	4	5
Our corporate culture **values and rewards flexibility, change, and continuous quality improvement with resources to support these values.**	1	2	3	4	5
When we make major decisions, we usually allow **enough time** to identify researchable questions and create/obtain, analyse, and consider research results and other evidence.	1	2	3	4	5

Questions 33-40

	Strongly disagree	Disagree	Neither agree nor disagree	Agree	Strongly agree
The CCG management team **evaluates the feasibility** of each option, including potential impact across the organisation as well as on clients, partners and other stakeholders.	1	2	3	4	5
Decision-makers in the CCG give **formal consideration to any recommendations** from staff who have developed or identified high-quality and relevant research.	1	2	3	4	5
Staff who have provided evidence and analysis usually **participate in decision-making discussions.**	1	2	3	4	5
Relevant **on-staff researchers are made part of decision-making discussions.**	1	2	3	4	5
Staff and appropriate stakeholders...					
... know **when and how major decisions will be made.**	1	2	3	4	5
... **contribute evidence and know how that information will be used.**	1	2	3	4	5
... receive **feedback** on decisions, with a rationale for the decision.	1	2	3	4	5
... are **informed of how available evidence influenced the choices** that were made in our CCG	1	2	3	4	5

Section 2 You and Research Evidence

This section focuses on your own, personal, use of research evidence. For each statement, please tick the **one** box that mirrors your view. Please be as honest as possible.

**Questions 41-43**	Strongly disagree	Disagree	Somewhat disagree	Neither agree nor disagree	Somewhat agree	Agree	Strongly agree
I **expect** to use research evidence to help think through what I will say or contribute to a CCG policy meeting.	☐	☐	☐	☐	☐	☐	☐
I **want** to use research evidence to help think through what I will say or contribute to a CCG policy meeting.	☐	☐	☐	☐	☐	☐	☐
I **intend** to use research evidence to help think through what I will say or contribute to a CCG policy meeting.	☐	☐	☐	☐	☐	☐	☐

44-47 Using research evidence to help think through what I will say or contribute to a CCG policy meeting is...

Very harmful	Moderately harmful	Slightly harmful	Neutral	Slightly beneficial	Moderately beneficial	Very beneficial
☐	☐	☐	☐	☐	☐	☐
Very bad	Moderately bad	Slightly bad	Neutral	Slightly good	Moderately good	Very good
☐	☐	☐	☐	☐	☐	☐
Very unpleasant (for me)	Moderately unpleasant (for me)	Slightly unpleasant (for me)	Neutral	Slightly pleasant (for me)	Moderately pleasant (for me)	Very pleasant (for me)
☐	☐	☐	☐	☐	☐	☐
Very unhelpful	Moderately unhelpful	Slightly unhelpful	Neutral	Slightly helpful	Moderately helpful	Very helpful
☐	☐	☐	☐	☐	☐	☐

48 **Most people who are important to me in my professional life think that...**

I should definitely not	I should almost certainly not	I should probably not	Neutral	I should probably	I should almost certainly	I should definitely
☐	☐	☐	☐	☐	☐	☐

... use research evidence to help think through what I will say or contribute to a CCG policy meeting.

Questions 49-52	Strongly disagree	Disagree	Somewhat disagree	Neither agree nor disagree	Somewhat agree	Agree	Strongly agree
**Those I work with expect me** to use research to help think through what I will say or contribute in a CCG policy meeting.	☐	☐	☐	☐	☐	☐	☐
**I feel under social pressure to use research evidence** to help think through what I will say or contribute in a CCG policy meeting.	☐	☐	☐	☐	☐	☐	☐
**People who are important to me in my professional life want me to use research evidence** to help think through what I will say or contribute in a CCG policy meeting.	☐	☐	☐	☐	☐	☐	☐
**I am confident that I could use research evidence** to help think through what I will say or contribute in a CCG policy meeting.	☐	☐	☐	☐	☐	☐	☐

Question 53-55	Very difficult	Moderately difficult	Slightly difficult	Neutral	Slightly easy	Moderately easy	Very easy
**For me to use research evidence** to help think through what I will say or contribute in a CCG policy meeting would be…	☐	☐	☐	☐	☐	☐	☐

	Strongly disagree	Disagree	Some-what disagree	Neither agree nor disagree	Some-what agree	Agree	Strongly agree
**The decision to use research evidence** to help think through what I will say or contribute in a CCG policy meeting is beyond my control.	☐	☐	☐	☐	☐	☐	☐
**Whether or not I use research evidence** to help think through what I will say or contribute in a CCG policy meeting **is entirely up to me.**	☐	☐	☐	☐	☐	☐	☐

Section 3 Your Relationship with Researchers

This section is interested in the relationships that you currently have with researchers *in general*. Circle the **single** number that represents your view.

Question 56-57

	Never	A few times a year	Once a month	Once a week	Every couple of days	Daily
How often do you meet face-to-face with researchers in the course of your job?	1	2	3	4	5	6
How often do you communicate via email, Skype, or telephone with researchers in the course of your job?	1	2	3	4	5	6

Question 58-62 In general, when you communicate (e.g., face to face or via email, phone, or Skype) with researchers, how do you find the contact? Please respond on each of the following scales. For example, on the first scale, if you find the contact *very friendly*, please circle number 7, or if you find the contact moderately *unfriendly*, you might circle 3.

**Not at all friendly**	1	2	3	4	5	6	7	**Very friendly**
**Not at all pleasant**	1	2	3	4	5	6	7	**Very pleasant**
**Not at all helpful**	1	2	3	4	5	6	7	**Very helpful**
**Not at all cooperative**	1	2	3	4	5	6	7	**Very cooperative**
**Very negative**	1	2	3	4	5	6	7	**Very positive**

Question 63-65 When you communicate with researchers, do you feel that this communication:

Helps me to achieve my goals?	**Not at all**	1	2	3	4	5	6	7	**Very much**
Helps researchers to achieve their goals?	**Not at all**	1	2	3	4	5	6	7	**Very much**
Helps researchers *and* me to achieve goals that benefit us both?	**Not at all**	1	2	3	4	5	6	7	**Very much**

Question 66-71 To what extent do you agree with each of the following statements?

*"In general, **CCGs** are very supportive of CCG leaders and researchers working closely together"*	**Not at all**	1	2	3	4	5	6	7	**Very much**
*"In general, the **NHS** is very supportive of CCG leaders and researchers working closely together"*	**Not at all**	1	2	3	4	5	6	7	**Very much**
"CCG leaders and researchers recognise the expertise of each others' group"	**Not at all**	1	2	3	4	5	6	7	**Very much**
"CCG leaders have a higher status than researchers in the NHS"	**Not at all**	1	2	3	4	5	6	7	**Very much**
"CCG leaders feel like part of one overarching team committed to achieving the same goals"	**Not at all**	1	2	3	4	5	6	7	**Very much**
"CCG leaders and researchers feel like members of two separate groups with different goals"	**Not at all**	1	2	3	4	5	6	7	**Very much**

Question 72-75 Next, we'd like to ask you about your position as a CCG leader.

"I identify strongly as a CCG leader"	**Not at all**	1	2	3	4	5	6	7	**Very much**
"Being a CCG leader is an important part of who I am"	**Not at all**	1	2	3	4	5	6	7	**Very much**
"I feel strong ties with other CCG leaders"	**Not at all**	1	2	3	4	5	6	7	**Very much**
"I feel a sense of solidarity with other CCG leaders"	**Not at all**	1	2	3	4	5	6	7	**Very much**

We would now like to ask you some questions about your general perceptions of researchers. Please answer as honestly as you can. Your responses are anonymous.

Questions 76-81 Based on your experience please rate the extent to which you have each of the following feelings about researchers *in general*. (Please circle one number on each scale).

Do you feel ...

Warm	1	2	3	4	5	6	7	***Cold***
Negative	1	2	3	4	5	6	7	***Positive***

Friendly	1	2	3	4	5	6	7	***Hostile***
Suspicious	1	2	3	4	5	6	7	***Trusting***
Respect	1	2	3	4	5	6	7	***Contempt***
Admiration	1	2	3	4	5	6	7	***Disgust***

Question Please use the scale to indicate your **overall attitude towards researchers by circling the bar on the scale that is closest to your feelings.**

Very negative

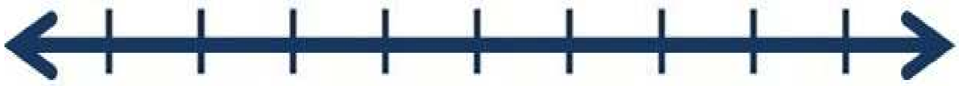

Very positive

Section 4 About You

Finally, we have a few questions about you. These are important as they will help us to understand different perspectives within clinical commissioning groups.

Question 83 In your current role with the CCG, do you have any formal responsibility for doing or managing research?

Yes - doing	☐	Yes - managing	☐
Neither	☐	Yes - both	☐

Question 84 What is your highest educational attainment?

School level (NVQ, GCSE, A Level or equivalent)	☐	Masters degree	☐
Undergraduate degree	☐	Higher degree (PhD)	☐

Question 85 Do you have any medical qualifications? If yes, please state.

No	☐	Yes	☐

…………………………………………………………………

…………………………………………………………………

Question 86 Do you have any previous experience of doing research? Tick as many as apply.

I have worked as a researcher in an academic context	I have commissioned research	I have been a co-applicant or advisor on a research project	I have been employed within a healthcare organisation as a researcher	Other – please give details below
☐	☐	☐	☐	☐

……

Question 87 When something new comes along I usually...

Like to be the first to take part and jump in with both feet	Start to take part fairly early on	Wait until some people have started doing it before I do	Start once the majority of other people are doing it	Wait until everyone else is doing it before having a go
☐	☐	☐	☐	☐

Question 87 Are you...

Male	☐
Female	☐

Thank you for completing this questionnaire. If you have any comments you would like to make on the topic of this survey, please use the box below.
